# Licorice extract inhibits the cGAS-STING pathway and protects against non-alcoholic steatohepatitis

**DOI:** 10.3389/fphar.2023.1160445

**Published:** 2023-04-04

**Authors:** Wei Luo, Guang Xu, Zheng Song, Wenqing Mu, Jincai Wen, Siwen Hui, Jia Zhao, Xiaoyan Zhan, Zhaofang Bai, Xiaohe Xiao

**Affiliations:** ^1^ School of Pharmacy, Chengdu University of Traditional Chinese Medicine, Chengdu, China; ^2^ Department of Hepatology, The Fifth Medical Center of PLA General Hospital, Beijing, China; ^4^ School of Traditional Chinese Medicine, Capital Medical University, Beijing, China; ^5^ Peking University 302 Clinical Medical School, Beijing, China; ^3^ Military Institute of Chinese Materia, Fifth Medical Center of Chinese PLA General Hospital, Beijing, China

**Keywords:** licorice extract, non-alcoholic steatohepatitis, anti-fibrosis, anti-inflammatory, cGAS-STING

## Abstract

**Background:** Inflammation and fibrosis are typical symptoms of non-alcoholic steatohepatitis (NASH), which is one of the most common chronic liver diseases. The cGAS-STING signaling pathway has been implicated in the progression of NASH, and targeting this pathway may represent a new therapeutic strategy. Licorice is a widely used herb with anti-inflammatory and liver-protective properties. In this study, we assessed the effect of licorice extract on the cGAS-STING pathway.

**Methods:** Bone marrow-derived macrophages (BMDMs) were treated with licorice extract and then stimulated with HT-DNA, 2'3'-cGAMP, or other agonists to activate the cGAS-STING pathway. Quantitative real-time PCR and western blot were conducted to analyze whether licorice extract could affect the cGAS-STING pathway. Methionine and choline-deficient diet (MCD) was used to induce NASH in mice, which were treated with licorice extract (500 mg/kg) by gavage and/or c-176 (15 mg/kg) by intraperitoneal injection every 2 days. After 6 weeks of treatment, histological analysis of liver tissue was performed, along with measurements of plasma biochemical parameters.

**Results:** Licorice extract inhibits cGAS-STING pathway activation. Mechanistically, it might function by inhibiting the oligomerization of STING. Treatment with licorice extract reduced inflammation and fibrosis in MCD diet-induced NASH mice models. Furthermore, we found that the therapeutic effect of combination treatment with licorice extract and C-176 (STING inhibitor) on the pathology and fibrosis of MCD diet-induced NASH models was similar to that of licorice extract or C-176 administered alone.

**Conclusion:** Licorice extract can inhibit the cGAS-STING pathway and improve hepatic inflammation and fibrosis in NASH mice models. It strongly suggests that licorice extract may be a candidate therapeutic for NASH.

## Introduction

Non-alcoholic steatohepatitis (NASH) is a severe and progressive form of non-alcoholic fatty liver disease (NAFLD), which can lead to cirrhosis and hepatocellular carcinoma (HCC) (Powell et al., 2021). Unfortunately, NASH has become increasingly prevalent in the past few decades, largely due to the high rates of metabolic syndrome, obesity, and type 2 diabetes in the population (Tilg et al., 2021). This trend has resulted in significant medical, economic, and social costs for both patients and the healthcare system (Younossi, 2019). Despite its high prevalence and growing impact on global health, no approved treatments are currently available to treat NASH (Sheka et al., 2020).

Stimulator of interferon genes (STING) is an innate immune protein that plays a significant role in the innate immune response (Ma and Damania, 2016). Increasing evidence has linked STING activation to various human diseases (Qiao et al., 2018). Cyclic GMP-AMP synthase (cGAS) is an upstream activator of STING. When abnormal DNA molecules appear in the cytoplasm, cGAS recognizes them and catalyzes the synthesis of the second messenger 2'3'-cyclic guanosine monophosphate (cGAMP). This further leads to the oligomerization of STING, causing its activation and entrance into the Golgi apparatus through the endoplasmic reticulum. The carboxyl terminus of STING recruits TANK-binding kinase 1 (TBK1), which phosphorylates STING and interferon regulatory factor 3 (IRF3) (Zhang et al., 2020). The activation of the nuclear factor-κB (NF-κB) transcription factor is regulated by STING-mediated IκB kinase (IKK) complex activation. As activated NF-κB and IRF3 enter the nucleus, they induce the expression of interferon and inflammatory cytokines, such as TNF-ɑ and IL-6 (Abe and Barber, 2014; Paul et al., 2021).

Recent studies have highlighted the crucial role of the cGAS-STING pathway in the progression of NASH. When hepatocytes are injured, they release mtDNA, which activates the cGAS-STING signaling pathway, triggering the expression of various inflammatory cytokines and chemokines (Arrese et al., 2016; Chen et al., 2021). Notably, liver steatosis, fibrosis, and inflammation were significantly reduced in STING-deficient mice fed with MCD or HFD diets, compared to wild-type (WT) mice (Yu et al., 2019). Kupffer cells (KCs), the resident macrophages of the liver, play a critical role in the development and progression of NASH (Tosello-Trampont et al., 2012). KCs are the first cells to respond to hepatocyte injury, leading to the activation of NF-κB and release of proinflammatory cytokines and chemokines, which could be attenuated by STING deficiency (Yu et al., 2019). Furthermore, STING expression was found to be increased in liver sections from NAFLD patients. Therefore, the cGAS-STING signaling pathway may be a key trigger of inflammatory responses in NASH progression and a potential new therapeutic target for NASH (Luo et al., 2018; Wang X. et al., 2020).

Licorice is a widely used Chinese herbal medicine known for its effectiveness in treating metabolic syndrome, asthma, and chronic liver diseases (Wang et al., 2013). It possesses various pharmacological properties, such as antiviral, anti-inflammatory, immunomodulatory, and hepatoprotective effects (Leite et al., 2022). Licorice is also a popular remedy for liver diseases in traditional Chinese medicine practiced in Japan and Europe (Li et al., 2019). In addition, licorice and its natural products are used to treat chronic viral hepatitis and other ailments (Li et al., 2019). Clinical trials have shown that licorice significantly reduces body weight and serum biochemical parameters, such as alanine aminotransferase (ALT) and aspartate transaminase (AST), in patients with metabolic syndrome and non-alcoholic fatty liver disease (Luís et al., 2018). Moreover, combining licorice extract with a low-calorie diet can improve lipid profiles and potentially help treat obesity-related fatty liver disease in obese patients (Alizadeh et al., 2018). Thus, licorice is a potent medicinal herb with anti-inflammatory and hepatoprotective effects.

In this study, we foung that licorice extract inhibited the HT-DNA-induced activation of the cGAS-STING pathway in BMDMs but did not affect the activation of the RIG-I-MAVS pathway triggered by poly (I: C). Mechanistically, licorice extract inhibited STING oligomerization, which suppressed its activation. In NASH mice models, licorice extract reduced liver inflammation and improved histological evaluation by inhibiting the STING pathway. These findings suggest that licorice may have therapeutic potential in treating liver diseases and inflammation.

## Method and reagents

### Animals

C57BL/6 mice of 6–8 weeks of age were purchased from Specific Pathogen-Free (SPF) Biotechnology Company Limited (Beijing, China). Random mice were selected for the study and grouped into specific sterile facilities. They were housed under controlled circumstances (12 h/12 h light/dark cycle). The Chinese People’s Liberation Army General Hospital’s Fifth Medical Centre’s Experimental Animal Welfare and Ethics Department approved all animal experiments.

### Cell culture

A femoral bone marrow sample of eight-week-old female C57BL/6 mice was collected for the isolation of bone marrow-derived macrophages (BMDMs). BMDMs were cultured in Dulbecco’s Modified Eagle’s Medium (DMEM) supplemented with 10% fetal bovine serum (FBS) and 1% penicillin/streptomycin (P/S). Human leukemic monocytes (THP-1 cells) were cultured in RPMI 1640 medium.

### Sample preparation and characterization

The licorice extract used in this study was purchased from Chengdu Despite Biotechnology Co., Ltd. The main source of the glycyrrhiza extract (powder) used in this study was distention Glycyrrhiza uralensis Fisch (Xinjiang), which was provided by Chengdu Desite Biotechnology Co., Ltd. Article No. DST20220215. The report of the identification of licorice is in Supplementary Figure S1.

After cleaning and grinding the raw material of licorice (Glycyrrhiza uralensis Fisch), 1,000 g of the herb were weighed and soaked in 70% ethanol for three extractions, using a herb to ethanol ratio of 1:20 by volume, and each extraction was carried out at 60°C for 4 h. The combined extract was concentrated to a specific gravity of approximately 1.1–1.2, and one-fifth of the resulting concentrate was dried to obtain 24 g of licorice extract. The cell administration method for licorice extract is prepared and used on demand. Each time, an appropriate amount of licorice extract is weighed and dissolved in OptiMEM medium (Gibco™), then filtered with a 0.22 µm filter before administration.

Liquid chromatography conditions are as follows: Sample: Licorice extract; Instrument: AB SCIEX X500 series QTOF Mass Spectrometer Column: XSelect HSS C18 (4.6 × 250 mm, 5 μm); Mobile phase: A: acetonitrile B: 0.1% formic acid/water, Elution conditions: 0 min, A:5%, B:95%; 3 min, A:16%, B:84%; 13 min, A:18%, B:82%; 18 min, A:25%, B:75%; 23 min, A:25%, B:75%; 43 min, A:37%, B:63%; 68min, A:95%, B:5%; Flow rate: 1.0 mL/min; Column temperature: 30°C; Sample size: 10 μL; Detection wavelength: 250 nm; Mass spectrometry detection method: Ion source: ESI ion source; Negative ion mode; Curtain gas: 35 psi; Gas 1:45 psi; Gas 2:45 psi; Temperature: 450°C; Ionization pressure: −4500 V, cluster removal voltage: −80 V; Full scanning range: m/z 80–1,500; Cracking voltage: −5 V. CE Spread: 0 V. The raw data of LC-MS was analyzed by MS-DIAL.

### Reagents and antibody

Polymyristate-13-acetate (PMA), Polyinosinic–polycytidylic acid (poly (I: C)), Herring testis (HT) DNA, and dimethyl sulfoxide (DMSO) were all purchased from Sigma-Aldrich (Jefferson City, United States). 2'3'-cGAMP were purchased from InvivoGen. Penicillin-streptomycin 100× sterile (CC004) was purchased from MacGene (Beijing, China). StarFect High-efficiency Transfection Reagents were purchased from GenStar. DMXAA (HY-10964). DiABZI STING agonist-1 trihydrochloride (HY-112921B) was purchased from Med Chem Express (State of New Jersey, US). Rabbit monoclonal anti-Phospho-IRF-3 (1:1000,86691) was purchased from Gene Tex (China). Rabbit monoclonal anti-Phospho-IRF-3 (1:1000,76439) was purchased from Abcam. TMEM173/STING Polyclonal antibody (1:2000,19851-1-AP), IRF3 Polyclonal antibody (1:2000,11312-1-AP), Alpha Tubulin Monoclonal antibody (1:1500, 66031-1-Ig) and Anti Lamin B (1:1500, 66095-1-Ig) antibodies were purchased from Proteintech (Chicago, United States).

### Cell counting Kit 8 assay

Cell viability was determined using the Cell Counting Kit 8 (CCK-8). We seeded BMDMs or THP-1 into 96-well plates at a density of 10^6^ cells/mL and incubated them overnight at 37°C. Different concentrations of licorice extract were used to treat the cells for 24 h. Cells were then incubated with CCK-8 reagent in a cell culture medium for 15 min. Optical density was measured at the wavelength of 450 nm after each incubation.

### Real-time RT-PCR

After the isolation of total RNA from indicated cells with TRIzol reagent (Invitrogen) as directed by the manufacturer’s instructions, reverse transcription was performed. Using SYBR GREEN MASTER MIX (Med Chem Express), gene transcripts were quantified by real-time PCR. The *ß*-actin was used as an internal control. A list of the primers that were used to amplify the target genes was presented in Supplementary Table S1.

### Enzyme-linked immunosorbent assay (ELISA)

The IFN-β levels in supernatants from cell cultures were measured using IFN-β Bioluminescent ELISA (luex-mifnbv2; InvivoGen) following the instructions for the assay kit.

### Western blotting

We detected the expression of p-IRF3, STING, and IRF3 in cell lysates by western blot analysis, using HSP90 as a loading control, as previously described (Wang Z. et al., 2020). Protein samples resolved on 10% SDS-PAGE were transferred to nitrocellulose membranes using a wet-transfer system and incubated overnight with primary antibodies at 4°C after pre-incubation with 5% fat-free milk for 1 h at room temperature. Horseradish peroxidase-labeled secondary antibodies was applied to the blots, and after washing with TBST, X-ray films were developed and fixed in a dark room for sensitivity.

### STING oligomerization assay

Assays for STING oligomerization were conducted as described previously (Li et al., 2018). In brief, a native sample buffer was used to load cell lysates on a native-PAGE gel, which was pre-run in an electrophoresis buffer (Cathode and anode chambers were respectively filled with 25 mM Tris-Cl, pH 8.4, 192 mM glycine with or without 5% deoxycholate.) for 30 min at 100 mA and then electrophoresed for 50 min at 25 mA. In the same way, as described above, SDS electrophoresis buffer (25 mM Tris pH 8.3, 250 mM Glycine, 0.1% SDS) was added to the gel and the gel was allowed to soak for 30 min at room temperature, followed by immunoblotting with an anti-STING antibody.

### Immunofluorescence

BMDMs or THP-1 cells were fixed with 4% paraformaldehyde for 15 min, permeabilized in 0.25% Triton X-100 with PBS for 20 min, and then blocked with 5% rapid blocking solution (Beijing, China, C200501) for 1 h. Afterward, the cells were stained with the indicated primary antibodies and then incubated with fluorescent-conjugated secondary antibodies. DAPI was used to counterstain the nuclei.

### MCD diet-induced NASH model

The study included feeding 6-week-old female C57BL/6 mice with either a methionine-choline-deficient (MCD, Dytz Biotechnology Co. Ltd.) or a methionine-choline-sufficient (MCS, Dytz Biotechnology Co. Ltd.) diet for 6 weeks. The model group was given the MCD diet, whereas the control group was given the MCS diet. At the same time, the treatment group received licorice extract dissolved in physiological saline (500 mg/kg to make a solution of 2 mg/mL, and no precipitation was observed) every 2 days, with the negative control group receiving physiological saline alone. The positive control group was administered with C-176 (15 mg/kg) alone by intraperitoneal injection every 2 days, and the combination group was given licorice extracts by gavage (500 mg/kg) combined with C-176 (15 mg/kg) by intraperitoneal injection for 6 weeks (n = 8 per group). Following anesthesia, the mice were euthanized, liver tissues were collected for mRNA analysis, and serum was separated for ALT and AST detection. Various histologic staining techniques were used to determine the collagen deposition and histopathology in mice liver tissues, including hematoxylin-eosin (HE), Masson, Sirius Red staining, and immumohistochemical staining.

### Statistical analyses

For the comparison of the two groups, an unpaired t-test was used. For the comparison of multiple groups, one-way ANOVA with Dunnett’s *post hoc* test or Kruskal–Wallis test was used, as appropriate (GraphPad Prism 6.0). The data were presented as mean ± SEM, and a *p*-value <0.05 was considered significant.

## Result

### Composition of licorice extract

Raw LC-MS data was imported into mass bank, inspect, and GNPs databases for comparison, including ingredient names, relative molecular mass, and peak area percentage. The peak identification spectrum was shown below. The compounds with qualitative results were listed in Table 1.

**TABLE 1:** Licorice extract inhibits DNA-triggered STING signaling pathway activation.

Number	Retention time	[M-H]-	MS/MS	Compound
1	15.05	549.1408	429.1073, 225.0678	Isoliquiritin apioside
2	15.80	417.1048	225.0677	Liquiritin
3	22.81	549.1422	429.1073, 225.0678	Celerose isoglyritin
4	24.39	417.1048	225.0677	Isoglycyrrhizin
5	25.10	267.0577	252.0411,195.0584	Formononetin
6	36.70	983.4125	821.4015, 351.0648	Glycyrrhizin A3
7	40.06	837.3605	351.0591	Glycyrrhizin G2
8	44.42	837.3587	351.0583	Hydroxyglycyrrhizic acid
9	47.66	821.3633	821.3966, 351.0579	Glycyrrhizic acid
10	49.42	821.3663	821.3983, 351.0588	Glycyrrhizin H2

### Licorice extract inhibits DNA-triggered STING signaling pathway activation

**FIGURE 1:**
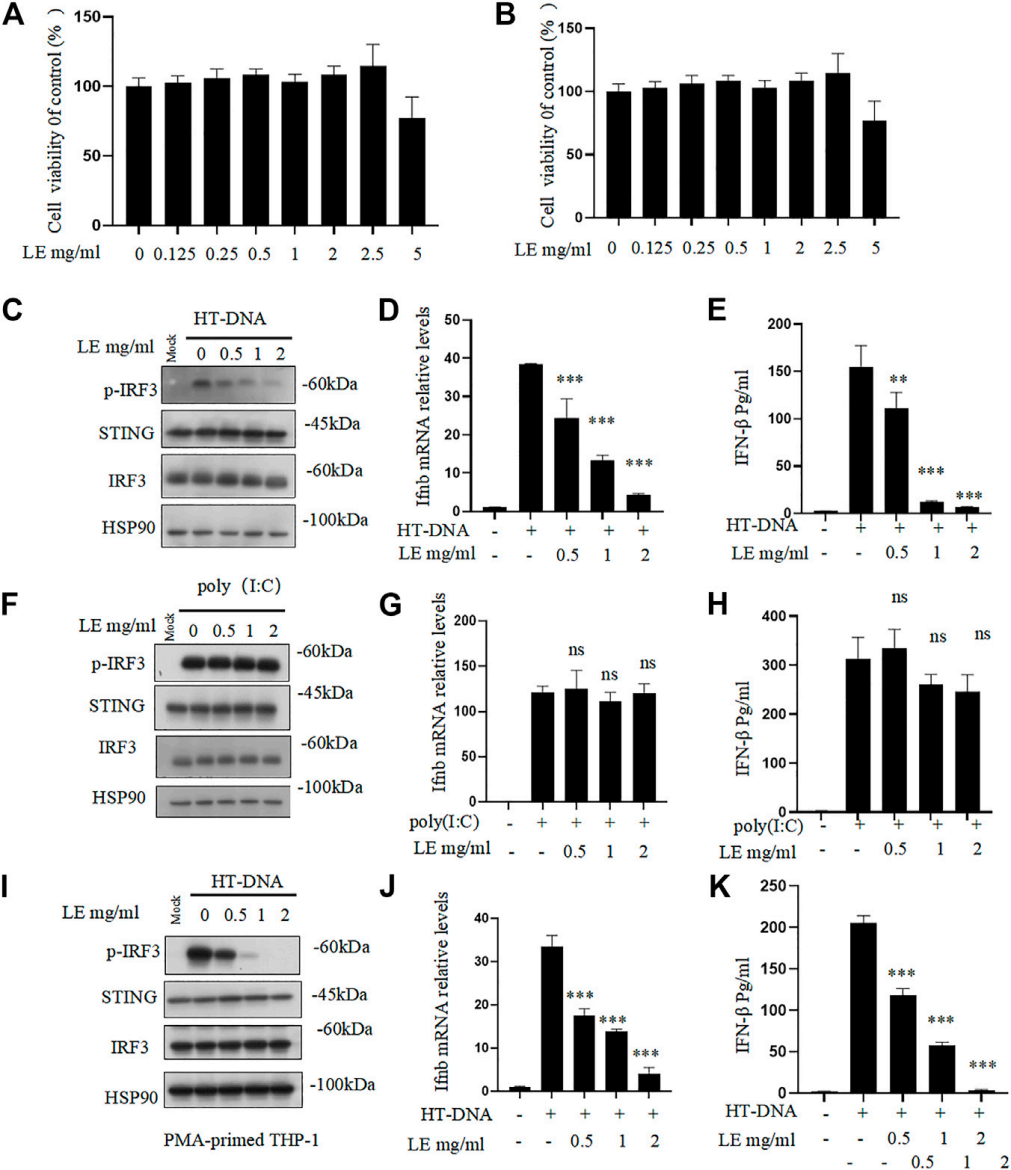
Licorice extract inhibits DNA-triggered STING signaling pathway activation. **(A, B)** The viability of BMDMs and THP-1 treated with different doses of licorice extract (LE) for 24 h was determined by using the Cell Counting Kit 8 (CCK-8). **(C)** BMDMs were pretreated for 1 h with various concentrations of LE and then transfected with HT-DNA. Using Western blotting, p-IRF3, STING, IRF3, and HSP90 were analyzed 2 h after HT-DNA transfection. **(D)** Quantitative PCR was performed to measure the Ifnb mRNA 4 h after HT-DNA transfection. **(E)** ELISA was performed to measure the amount of IFN-β protein secreted in cell culture supernatants 12 h after HT-DNA transfection. **(F)** BMDMs were pretreated for 1 h with various concentrations of LE and then transfected with poly (I: C). Using Western blotting, p-IRF3, STING, IRF3, and HSP90 were analyzed 2 h after poly (I: C) transfection. **(G)** Quantitative PCR was performed to measure the Ifnb mRNA 4 h after poly (I: C) transfection. **(H)** ELISA was performed to measure the amount of IFN-β protein secreted in cell culture supernatants 12 h after poly (I: C) transfection. **(I)** THP-1 was pretreated for 1 h with various concentrations of LE and then transfected with HT-DNA. Using Western blotting, p-IRF3, STING, IRF3, and HSP90 were analyzed 2 h after HT-DNA transfection. **(J)** Quantitative PCR was performed to measure the Ifnb mRNA 4 h after HT-DNA transfection. **(K)** ELISA was performed to measure the amount of IFN-β protein secreted in cell culture supernatants 12 h after HT-DNA transfection. Statistical differences were analyzed by One-way ANOVA with Dunnett’s *post hoc* test. Data information: Error bars represent the mean ± SEM of three technical replicates. *p < 0.05; **p < 0.001; ***p < 0.001; ns, not significant.

Licorice extract was initially tested for its cytotoxicity. Different concentrations of the licorice extract were applied to BMDMs and THP-1 cells for 24 h, after which the viability of the cells was determined. The results indicated that the licorice extract did not have a significant cytotoxic effect on BMDMs and THP-1 cells at concentrations lower than 2.5 mg/mL (Figures 1A,B). To further investigate the effect of licorice extract on the activation of the cGAS-STING signaling pathway, BMDMs were pre-treated with various concentrations of licorice extract and then stimulated with HT-DNA (transfected into the cell) to activate the cGAS-STING pathway. The results showed that licorice extract inhibited HT-DNA-induced phosphorylation of IRF3, and a dose-dependent effect could be observed (Figure 1C). RT-qPCR analysis was performed to determine the expression levels of the Ifnb gene. It was found that licorice extract could inhibit the induction of Ifnb mRNA caused by HT-DNA (Figure 1D). The ELISA results suggested that the licorice extract suppressed IFN-β secretion (Figure 1E). Moreover, STING activation also initiates the NF-κB signaling pathway *via* IKK, which phosphorylates p65. Phosphorylated p65 enters the nucleus and leads to the expression of pro-inflammatory cytokines. Licorice extract also significantly inhibited the upregulation of HT-DNA-induced phosphorylated p65 and mRNA expression of TNF-α and IL-6 in BMDM cells (Supplementary Figures S2A–C).

However, our results showed that licorice extract did not affect cytoplasmic poly (I: C)-triggered phosphorylation of IRF3 (Figure 1F), Ifnb mRNA expression (Figure 1G), and IFN-β secretion (Figure 1H), which are mediated by RIG-I-MAVS pathway (Chiu et al., 2009). This suggests that licorice extract does not affect this pathway. The inhibitory effect of licorice extract on the cGAS-STING pathway was also confirmed in human leukemia monocytic cell line THP-1 (Figures 1I–K). The quantitative results of p-IRF3 protein also showed (Supplementary Figures S2D–F). These data indicate that licorice extract inhibits the HT-DNA-induced cGAS-STING pathway.

### The licorice extract inhibits STING-dependent signal transduction

**FIGURE 2:**
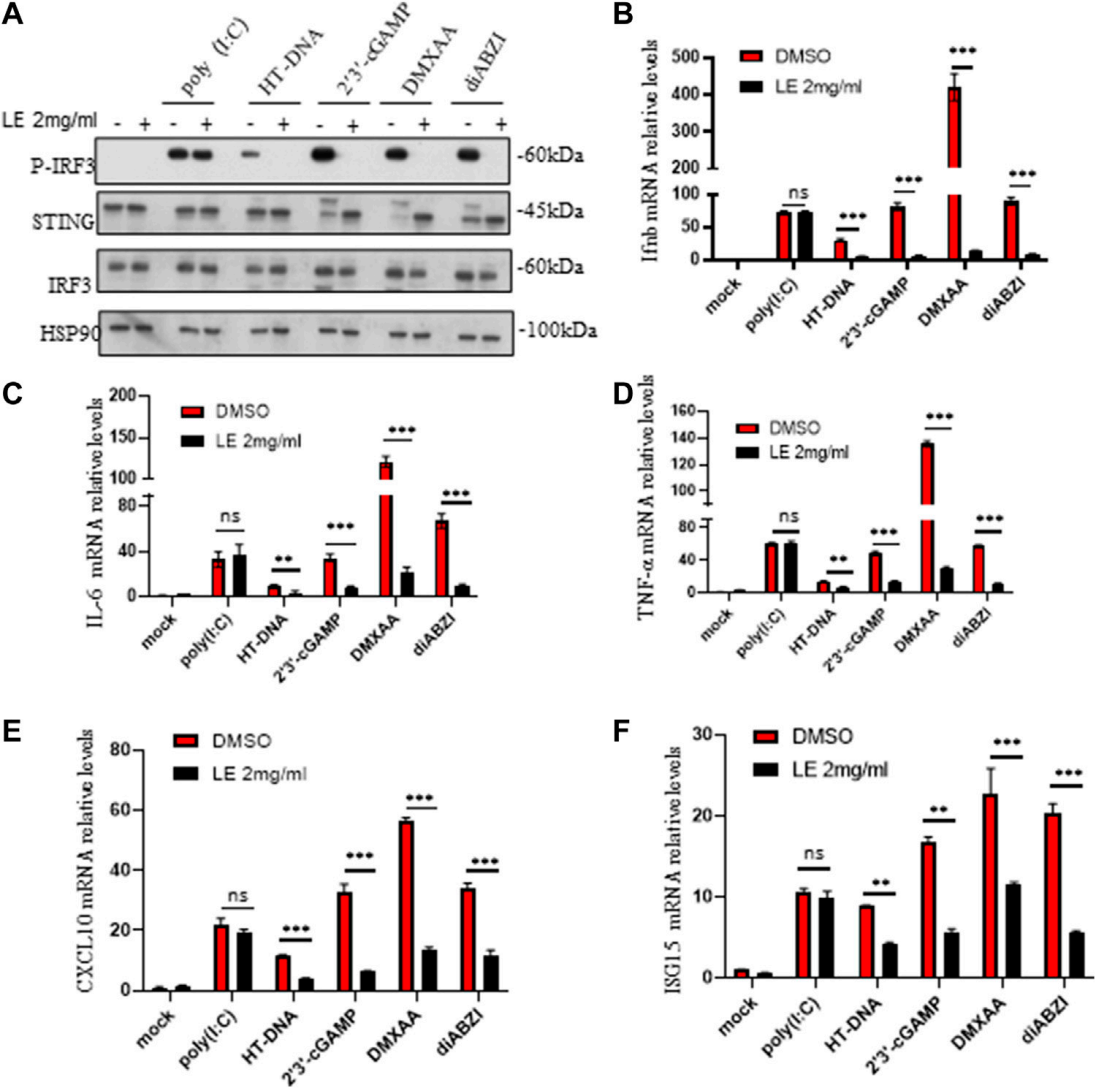
The licorice extract inhibits STING-dependent signal transduction. **(A)** BMDMs were pretreated for 1 h with LE and then stimulated with HT-DNA, poly (I:C), 2'3'-cGAMP, DMXAA, and diABZI for 2 h. Whole cell lysates were analyzed using Western blotting for p-IRF3, STING, IRF3, and HSP90. **(B–F)** The induction of Ifnb, TNF-α, IL-6, cxcl10, and ISG15 mRNAs was measured by quantitative PCR 4 h later. Data information: Error bars represent the mean ± SEM of three technical replicates. Statistical differences were analyzed by unpaired t-test, with *p < 0.05, **p < 0.001, ***p < 0.001, and ns, indicating not significant.

To further confirm our findings, we investigated the effects of licorice extract on the activation of the STING signaling pathway induced by various agonists, including HT-DNA, 2'3'-cGAMP, DMXAA (Gao et al., 2013), and diABZI (Ramanjulu et al., 2018), in BMDM cells. Western blot analysis showed that licorice extract reduced the phosphorylation of IRF3 induced by these stimuli (Figure 2A). The protein quantification results also showed that licorice extract inhibited the expression of p-IRF3 protein (Supplementary Figure S2G). Additionally, licorice extract significantly inhibited the upregulation of Ifnb, TNF-α, IL-6, CXCL10, and ISG15 mRNA expression in BMDMs induced by these STING agonists (Figures 2B–F). However, Poly (I:C)-triggered RIG-I-mediated signaling was not affected by licorice extract (Figures 2A–F). Our results suggest that licorice extract can specifically inhibit the activation of the STING signaling pathway.

### Licorice extract inhibits oligomerization of STING

**FIGURE 3:**
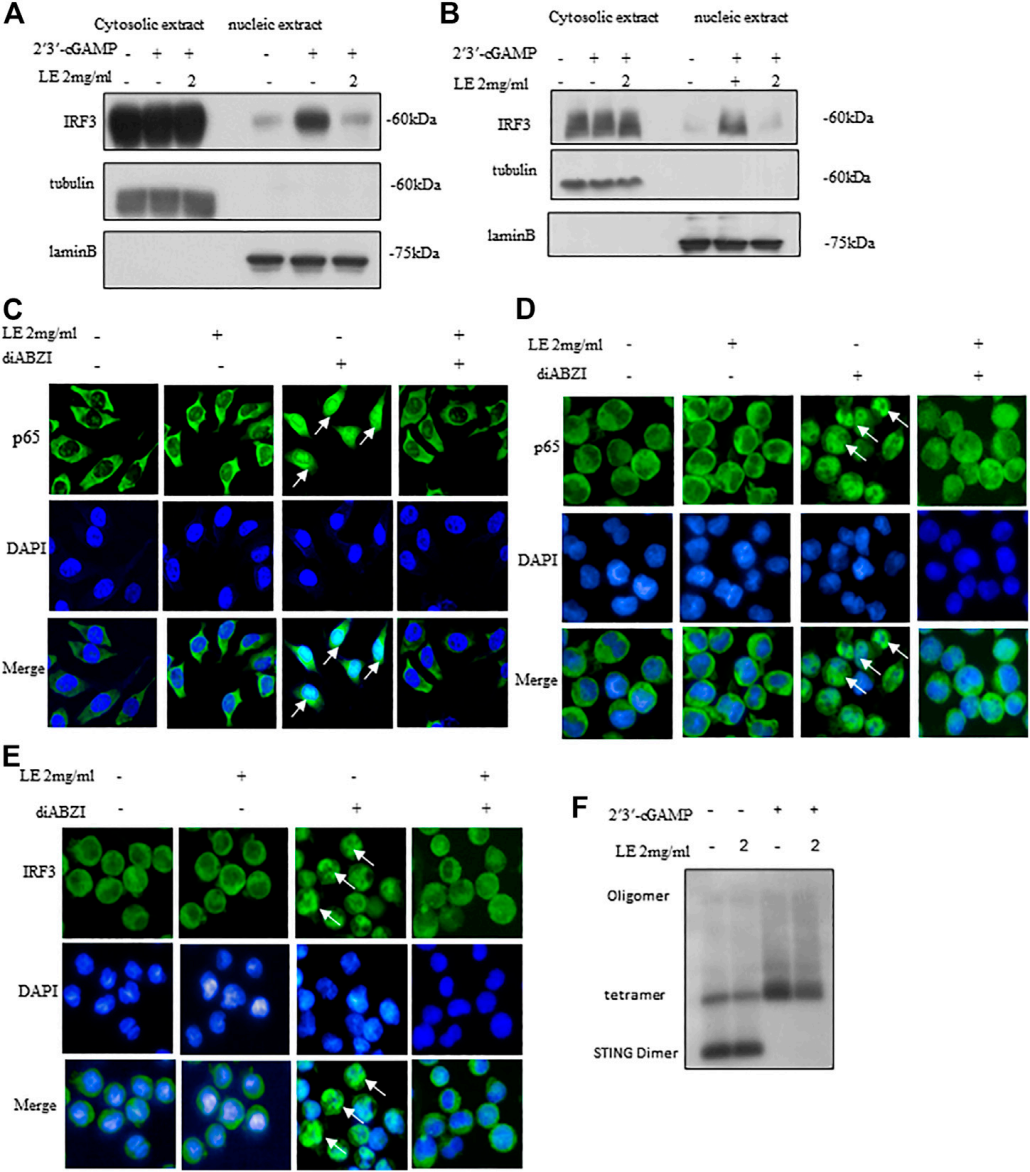
Licorice extract inhibits oligomerization of STING. **(A, B)** The BMDMs or THP-1 cells were pretreated for 1 h with LE and then transfected with 2′3′-cGAMP. The indicated antibodies were used to immunoblot the cytoplasmic and nuclear fractions. Fractional accuracy was assessed based on the levels of LaminB and Tubulin. **(C)** Immunofluorescence was used to visualize BMDMs pretreated or not with LE (2 mg/mL), stimulated for 2 h with diABZI, and immunostained with anti-p65 antibodies (Scale bars, 25 μm), Image by high-content analysis (Thermo Scientific HCA). **(D, E)** THP-1, pretreated or not with LE (2 mg/mL), was stimulated with diABZI for 2 h and immunostained with anti-p65 and anti-IRF3 (Scale bars, 25 μm), Image by high-content analysis (Thermo Scientific HCA). **(F)** After pretreatment with LE, BMDMs were stimulated with 2′3′-cGAMP for 1 h and immunoblots were used to analyze STING oligomerization.

Upon activation of STING, it undergoes phosphorylation by TBK1, which triggers a kinase cascade leading to the phosphorylation of IRF3 and p65, promoting their nuclear translocation (Bai and Liu, 2019). In our study, licorice extract significantly reduced the nuclear translocation of IRF3 induced by 2'3'-cGAMP (Figures 3A,B). Our IRF protein quantification results further showed that nuclear translocation of IRF3 was reduced following treatment with licorice extract (Supplementary Figures S2H–I). To confirm the inhibitory effect of licorice extract on STING activation, we used immunofluorescence staining to examine the nuclear translocation of phosphorylated p65 transcription factors stimulated by diABZI in BMDM cells and THP-1 cells (Figures 3C,D). Moreover, we found that licorice extract reduced diABZI-induced nuclear translocation of IRF3 in THP-1 cells (Figure 3E).

Considering the hierarchical relationship of these signaling molecules, we proposed that licorice extract may be involved in the regulation of the STING signalosome. STING oligomerization is critical for the activation of TBK1 as it can induce the trans-autophosphorylation of adjacent TBK1 molecules (Bai and Liu, 2019; Zhang et al., 2019). Upon binding to 2'3'-cGAMP, STING becomes oligomerized, which activates downstream signaling pathways (Zhang et al., 2019). Our result showed that licorice extract inhibited STING oligomerization induced by 2'3'-cGAMP (Figure 3F) and our quantitative results are also support this observation (Supplementary Figure S2J). In summary, licorice extract inhibited the nuclear translocation of IRF3 and p65, suggesting that it inhibits STING oligomerization as a potential mechanism of action.

### Licorice extracts reduced hepatocyte inflammation and fibrosis associated with NASH

Inhibition of STING pathway activation reduces liver inflammation and fibrosis, as well as ameliorates the pathological characteristics of NASH (Yu et al., 2019). It has been shown that C-176 (Haag et al., 2018) a small molecule inhibitor of STING, alleviates the autoinflammatory diseases caused by STING pathway in mice. To validate that licorice extract inhibited the progression of NASH *via* the STING pathway, C-176 was used as a positive control.

**FIGURE 4:**
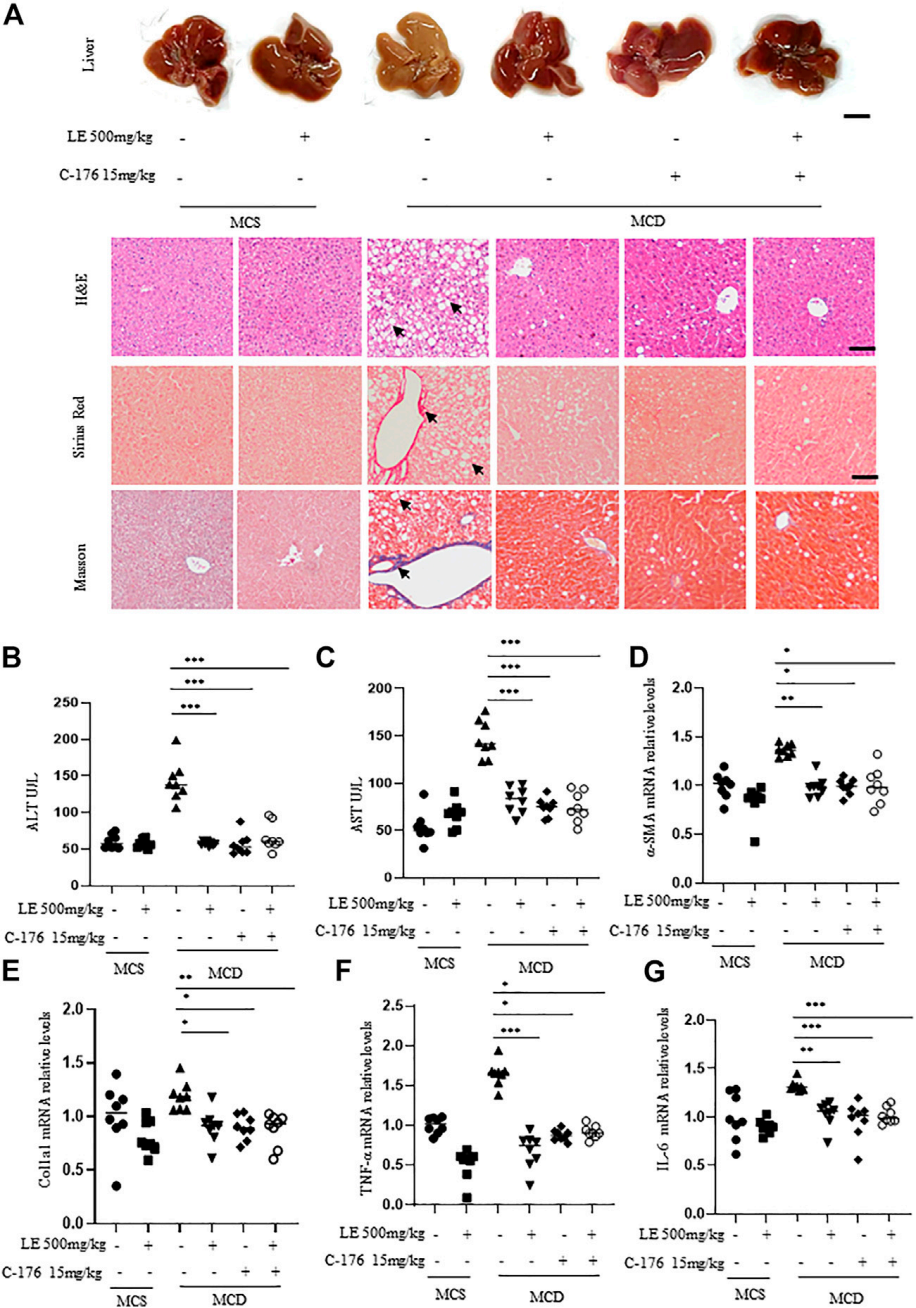
Licorice extract exhibits therapeutic effects in the NASH model. **(A)** 6 weeks old female C57BL/6 mice were continuously fed MCS or MCD diets for 6 weeks under the same growth conditions. At the same time, they were administered with licorice extract by gavage, injected intraperitoneally with C-176 (15 mg/kg), or treated with a combination of licorice extract and C-176 every 2 days (n = 8 mice per group). Representative liver images and histological images of Hematoxylin-eosin (H&E), Sirius Red, and Masson’s Trichrome stained liver tissue sections are shown. Scale bar: 0.5 cm (top row) and 100 um (3 bottom rows). **(B, C)** The serum levels of ALT and AST (n = 8 mice per group) were measured using ELISA. **(D–G)** Quantitative real-time PCR analysis was used to detect the mRNA levels of *a*-SMA **(D)**, Col1a1 **(E)**, TNF-α **(F)**, and IL-6 **(G)** in the livers of mice (n = 8 per group). Data information: Error bars represent mean ± SEM. *p < 0.05; **p < 0.001; ***p < 0.001; ns, not significant. (Statistical differences were analyzed by one-way ANOVA with Dunnett’s *post hoc* test or Kruskal–Wallis test).

The liver morphology of mice fed with MCD, which mimics the human NASH, differed significantly from that of mice fed with MCS. These differences included the presence of steatosis, ballooning, and fibrosis (Figure 4A), as well as significantly increased serum ALT (Figure 4B) and AST (Figure 4C) levels. However, treatment with licorice extract significantly improved the disease phenotype (Figures 4A–C). We also conducted MCD animal liver HE pathological scoring (Supplementary Table S2) and collagen fiber quantitative analysis (Supplementary Figure S3A–B). The results showed that licorice extract significantly inhibited liver fibrosis in MCD mice.

α-SMA (alpha-smooth muscle actin) and Col1a1 (type I collagen alpha 1 chain) are common indicators for assessing the degree and progression of liver fibrosis in NASH (Anstee et al., 2019). Immunohistochemistry results showed that licorice extract can inhibit the protein expression of *a*-SMA (Supplementary Figure S3C), and at the same time, licorice extract can also suppress the expression levels of *a*-SMA (Figure 4D) and Col1a1 (Figure 4E) genes. Moreover, it was found to suppress the pro-inflammatory genes TNF-α (Figure 4F) and IL-6 mRNA (Figure 4G) in MCD mice. This suggests that licorice extract has a suppressive effect on hepatic stellate cell activation, liver fibrosis, and the inflammatory response in NASH models. We used western blot to detect cGAS-STING related proteins in liver tissue, and found that p-IRF3 expression was elevated in MCD mice. After treatment with licorice extract, p-IRF3 protein was decreased (Supplementary Figure S3D–E).

In addition, compared to the negative control group, the disease phenotype was significantly alleviated or improved after treatment with C-176 (Figure 4A). C-176 treatment inhibited their serum ALT and AST levels, *a*-SMA and Col1a1 mRNA expression (Figures 4B–E) as well as the levels of pro-inflammatory genes including TNF-α and IL-6 (Figures 4F,G). Lastly, we also found that the therapeutic effect of combination treatment on fibrosis in the MCD diet-induced NASH model was similar to that of licorice extract or C-176 alone (Figures 4A–G).

Taken together, these results indicate that licorice extract alleviated liver inflammation and improved NASH pathology by inhibiting the activation of the STING pathway in mice models of NASH.

## Discussion

Our study found that licorice extract inhibited the activation of the cGAS-STING pathway induced by HT-DNA and disrupted STING oligomerization during activation. Furthermore, in mouse models of NASH, the extract reduced histological inflammation and hepatic fibrosis, possibly by inhibiting cGAS-STING pathway activation.

Licorice is a commonly used herb in traditional medicine, and previous studies have suggested its protective effect in inflammatory diseases (Wang et al., 2015). The effect of licorice and its components on NASH has also been investigated (Li et al., 2019; Wahab et al., 2021). For example, one study found that administering 1,000 mg of licorice extract per day for 12 weeks significantly improved plasma liver enzyme levels, glycemic index, oxidative stress parameters, and liver steatosis in 60 NAFLD patients (Rostamizadeh et al., 2022). Although licorice extract has also been reported to inhibit the expression levels of proinflammatory cytokines, such as TNF-α, IL-1β, and IL-6 (Kim et al., 2006; Yu et al., 2015; Yang et al., 2017; Frattaruolo et al., 2019; El-Saber Batiha et al., 2020), the mechanism underlying its protective effect in NASH remains incompletely understood. Our study showed that licorice extract suppressed the cGAS-STING pathway to reduce inflammation and alleviate NASH, providing new insight into the mechanism of its therapeutic effect in NASH.

In this study, we found that licorice extract specifically regulated the cGAS-STING pathway and did not affect poly (I:C)-triggered RIG-I-mediated signaling activation in BMDMs (Figures 1C–K). The inhibitory effect of licorice extract was further validated by the administration of 2'3'-cGAMP, DMXAA, and diABZI on BMDMs (Figure 2). The cGAMP stimulation leads to the oligomerization of STING and enhances the binding between STING and TBK1. At the same time, STING oligomerization induced by cGAMP also provided conditions for the phosphorylation of STING by TBK1 (Zhang et al., 2019). In our study, we found that licorice extract affected the oligomerization of STING (Figure 3F), but further research is required on its specific targets and active ingredients.

The cGAS-STING pathway plays a vital role in the development of NASH (Wang X. et al., 2020). Our research has found that licorice extract inhibits the activation of the cGAS-STING signaling pathway by suppressing the oligomerization of STING. C-176 is an inhibitor of STING that interferes with downstream signaling by inhibiting STING palmitoylation (Haag et al., 2018). Additionally, C-176 can improve inflammation and autoimmune diseases caused by abnormal activation of the cGAS-STING signaling pathway (Pham et al., 2021; Ma et al., 2022; Wu et al., 2022).

In the MCD diet-induced NASH mice model, results showed that both licorice extract and C-176 (a STING inhibitor) treatment significantly protected against the MCD diet-induced liver morphological changes, hepatic steatosis, ballooning, and fibrosis (Figure 4A). In addition, both treatments decreased the serum levels of ALT and AST (Figures 4B,C) and inhibited the expression of *a*-SMA and Col1a1 mRNA (Figures 4D,E) as well as the levels of pro-inflammatory genes including TNF-α and IL-6 (Figures 4F,G). However, the protective effect of licorice extract plus C-176 treatment was not significantly better than the groups of licorice extract or C-176 treatment alone. Taken together, these results indicate that licorice extract can reverse the pathological process of MCD-induced NASH by attenuating the STING pathway activation.

## Conclusion

In conclusion, our study suggests that licorice extract, which blocks the cGAS-STING pathway activation *via* suppressing STING oligomerization, may serve as a potential drug for the treatment of NASH and other related inflammatory diseases mediated by cGAS-STING signaling pathway.

## Data Availability

The raw data supporting the conclusions of this article will be made available by the authors, without undue reservation.

## References

[B1] Abe T. Barber G. N. (2014). Cytosolic-DNA-mediated, STING-dependent proinflammatory gene induction necessitates canonical NF-κB activation through TBK1. J. Virol. 88, 5328–5341. 10.1128/JVI.00037-14 24600004 PMC4019140

[B2] Alizadeh M. Namazi N. Mirtaheri E. Sargheini N. Kheirouri S. (2018). Changes of insulin resistance and adipokines following supplementation with Glycyrrhiza glabra L. Extract in combination with a low-calorie diet in overweight and obese subjects: A randomized double blind clinical trial. Adv. Pharm. Bull. 8, 123–130. 10.15171/apb.2018.015 29670847 PMC5896387

[B3] Anstee Q. M. Reeves H. L. Kotsiliti E. Govaere O. Heikenwalder M. (2019). From NASH to HCC: Current concepts and future challenges. Nat. Rev. Gastroenterol. Hepatol. 16, 411–428. 10.1038/s41575-019-0145-7 31028350

[B4] Arrese M. Cabrera D. Kalergis A. M. Feldstein A. E. (2016). Innate immunity and inflammation in NAFLD/NASH. Dig. Dis. Sci. 61, 1294–1303. 10.1007/s10620-016-4049-x 26841783 PMC4948286

[B5] Bai J. Liu F. (2019). The cGAS-cGAMP-STING pathway: A molecular link between immunity and metabolism. Diabetes 68, 1099–1108. 10.2337/dbi18-0052 31109939 PMC6610018

[B6] Chen C. Yang R. X. Xu H. G. (2021). STING and liver disease. J. Gastroenterol. 56, 704–712. 10.1007/s00535-021-01803-1 34159442 PMC8219471

[B7] Chiu Y. H. Macmillan J. B. Chen Z. J. (2009). RNA polymerase III detects cytosolic DNA and induces type I interferons through the RIG-I pathway. Cell. 138, 576–591. 10.1016/j.cell.2009.06.015 19631370 PMC2747301

[B8] El-Saber Batiha G. Magdy Beshbishy A. El-Mleeh A. Abdel-Daim M. M. Prasad Devkota H. (2020). Traditional uses, bioactive chemical constituents, and pharmacological and toxicological activities of Glycyrrhiza glabra L. (Fabaceae). Biomolecules 10 (3), 352. 10.3390/biom10030352 32106571 PMC7175350

[B9] Frattaruolo L. Carullo G. Brindisi M. Mazzotta S. Bellissimo L. Rago V. (2019). Antioxidant and anti-inflammatory activities of flavanones from Glycyrrhiza glabra L. (licorice) leaf phytocomplexes: Identification of licoflavanone as a modulator of NF-kB/MAPK pathway. Antioxidants (Basel) 8, 186. 10.3390/antiox8060186 31226797 PMC6616548

[B10] Gao P. Ascano M. Zillinger T. Wang W. Dai P. Serganov A. A. (2013). Structure-function analysis of STING activation by c[G(2',5')pA(3',5')p] and targeting by antiviral DMXAA. Cell. 154, 748–762. 10.1016/j.cell.2013.07.023 23910378 PMC4386733

[B11] Haag S. M. Gulen M. F. Reymond L. Gibelin A. Abrami L. Decout A. (2018). Targeting STING with covalent small-molecule inhibitors. Nature 559, 269–273. 10.1038/s41586-018-0287-8 29973723

[B12] Kim J. K. Oh S. M. Kwon H. S. Oh Y. S. Lim S. S. Shin H. K. (2006). Anti-inflammatory effect of roasted licorice extracts on lipopolysaccharide-induced inflammatory responses in murine macrophages. Biochem. Biophys. Res. Commun. 345, 1215–1223. 10.1016/j.bbrc.2006.05.035 16716255

[B13] Leite C. D. S. Bonafé G. A. Carvalho Santos J. Martinez C. a. R. Ortega M. M. Ribeiro M. L. (2022). The anti-inflammatory properties of licorice (Glycyrrhiza glabra)-derived compounds in intestinal disorders. Int. J. Mol. Sci. 23, 4121. 10.3390/ijms23084121 35456938 PMC9025446

[B14] Li S. Hong Z. Wang Z. Li F. Mei J. Huang L. (2018). The cyclopeptide astin C specifically inhibits the innate immune CDN sensor STING. Cell. Rep. 25, 3405–3421.e7. 10.1016/j.celrep.2018.11.097 30566866

[B15] Li X. Sun R. Liu R. (2019). Natural products in licorice for the therapy of liver diseases: Progress and future opportunities. Pharmacol. Res. 144, 210–226. 10.1016/j.phrs.2019.04.025 31022523

[B16] Luís Â. Domingues F. Pereira L. (2018). Metabolic changes after licorice consumption: A systematic review with meta-analysis and trial sequential analysis of clinical trials. Phytomedicine 39, 17–24. 10.1016/j.phymed.2017.12.010 29433679

[B17] Luo X. Li H. Ma L. Zhou J. Guo X. Woo S. L. (2018). Expression of STING is increased in liver tissues from patients with NAFLD and promotes macrophage-mediated hepatic inflammation and fibrosis in mice. Gastroenterology 155, 1971–1984.e4. 10.1053/j.gastro.2018.09.010 30213555 PMC6279491

[B18] Ma X. M. Geng K. Law B. Y. Wang P. Pu Y. L. Chen Q. (2022). Lipotoxicity-induced mtDNA release promotes diabetic cardiomyopathy by activating the cGAS-STING pathway in obesity-related diabetes. Cell. Biol. Toxicol. 10.1007/s10565-021-09692-z PMC10042943 35235096

[B19] Ma Z. Damania B. (2016). The cGAS-STING defense pathway and its counteraction by viruses. Cell. Host Microbe 19, 150–158. 10.1016/j.chom.2016.01.010 26867174 PMC4755325

[B20] Paul B. D. Snyder S. H. Bohr V. A. (2021). Signaling by cGAS-STING in neurodegeneration, neuroinflammation, and aging. Trends Neurosci. 44, 83–96. 10.1016/j.tins.2020.10.008 33187730 PMC8662531

[B21] Pham P. T. Fukuda D. Nishimoto S. Kim-Kaneyama J. R. Lei X. F. Takahashi Y. (2021). STING, a cytosolic DNA sensor, plays a critical role in atherogenesis: A link between innate immunity and chronic inflammation caused by lifestyle-related diseases. Eur. Heart J. 42, 4336–4348. 10.1093/eurheartj/ehab249 34226923

[B22] Powell E. E. Wong V. W. Rinella M. (2021). Non-alcoholic fatty liver disease. Lancet 397, 2212–2224. 10.1016/S0140-6736(20)32511-3 33894145

[B23] Qiao J. T. Cui C. Qing L. Wang L. S. He T. Y. Yan F. (2018). Activation of the STING-IRF3 pathway promotes hepatocyte inflammation, apoptosis and induces metabolic disorders in nonalcoholic fatty liver disease. Metabolism 81, 13–24. 10.1016/j.metabol.2017.09.010 29106945

[B24] Ramanjulu J. M. Pesiridis G. S. Yang J. Concha N. Singhaus R. Zhang S. Y. (2018). Design of amidobenzimidazole STING receptor agonists with systemic activity. Nature 564, 439–443. 10.1038/s41586-018-0705-y 30405246

[B25] Rostamizadeh P. Asl S. Far Z. G. Ahmadijoo P. Mahmudiono T. Bokov D. O. (2022). Effects of licorice root supplementation on liver enzymes, hepatic steatosis, metabolic and oxidative stress parameters in women with nonalcoholic fatty liver disease: A randomized double-blind clinical trial. Phytother. Res. 36, 3949–3956. 10.1002/ptr.7543 35785498

[B26] Sheka A. C. Adeyi O. Thompson J. Hameed B. Crawford P. A. Ikramuddin S. (2020). Nonalcoholic steatohepatitis: A review. Jama 323, 1175–1183. 10.1001/jama.2020.2298 32207804

[B27] Tilg H. Adolph T. E. Dudek M. Knolle P. (2021). Non-alcoholic fatty liver disease: The interplay between metabolism, microbes and immunity. Nat. Metab. 3, 1596–1607. 10.1038/s42255-021-00501-9 34931080

[B28] Tosello-Trampont A. C. Landes S. G. Nguyen V. Novobrantseva T. I. Hahn Y. S. (2012). Kuppfer cells trigger nonalcoholic steatohepatitis development in diet-induced mouse model through tumor necrosis factor-α production. J. Biol. Chem. 287, 40161–40172. 10.1074/jbc.M112.417014 23066023 PMC3504730

[B29] Wahab S. Annadurai S. Abullais S. S. Das G. Ahmad W. Ahmad M. F. (2021). Glycyrrhiza glabra (licorice): A comprehensive review on its phytochemistry, biological activities, clinical evidence and toxicology. Plants (Basel) 10, 2751. 10.3390/plants10122751 34961221 PMC8703329

[B30] Wang L. Yang R. Yuan B. Liu Y. Liu C. (2015). The antiviral and antimicrobial activities of licorice, a widely-used Chinese herb. Acta Pharm. Sin. B 5, 310–315. 10.1016/j.apsb.2015.05.005 26579460 PMC4629407

[B31] Wang X. Rao H. Zhao J. Wee A. Li X. Fei R. (2020). STING expression in monocyte-derived macrophages is associated with the progression of liver inflammation and fibrosis in patients with nonalcoholic fatty liver disease. Lab. Invest. 100, 542–552. 10.1038/s41374-019-0342-6 31745210

[B32] Wang X. Zhang H. Chen L. Shan L. Fan G. Gao X. (2013). Liquorice, a unique "guide drug" of traditional Chinese medicine: A review of its role in drug interactions. J. Ethnopharmacol. 150, 781–790. 10.1016/j.jep.2013.09.055 24201019

[B33] Wang Z. Xu G. Wang H. Zhan X. Gao Y. Chen N. (2020). Icariside Ⅱ, a main compound in Epimedii Folium, induces idiosyncratic hepatotoxicity by enhancing NLRP3 inflammasome activation. Acta Pharm. Sin. B 10, 1619–1633. 10.1016/j.apsb.2020.03.006 33088683 PMC7564030

[B34] Wu B. Xu M. M. Fan C. Feng C. L. Lu Q. K. Lu H. M. (2022). STING inhibitor ameliorates LPS-induced ALI by preventing vascular endothelial cells-mediated immune cells chemotaxis and adhesion. Acta Pharmacol. Sin. 43, 2055–2066. 10.1038/s41401-021-00813-2 34907359 PMC9343420

[B35] Yang R. Yuan B. C. Ma Y. S. Zhou S. Liu Y. (2017). The anti-inflammatory activity of licorice, a widely used Chinese herb. Pharm. Biol. 55, 5–18. 10.1080/13880209.2016.1225775 27650551 PMC7012004

[B36] Younossi Z. M. (2019). Non-alcoholic fatty liver disease - a global public health perspective. J. Hepatol. 70, 531–544. 10.1016/j.jhep.2018.10.033 30414863

[B37] Yu J. Y. Ha J. Y. Kim K. M. Jung Y. S. Jung J. C. Oh S. (2015). Anti-Inflammatory activities of licorice extract and its active compounds, glycyrrhizic acid, liquiritin and liquiritigenin, in BV2 cells and mice liver. Molecules 20, 13041–13054. 10.3390/molecules200713041 26205049 PMC6332102

[B38] Yu Y. Liu Y. An W. Song J. Zhang Y. Zhao X. (2019). STING-mediated inflammation in Kupffer cells contributes to progression of nonalcoholic steatohepatitis. J. Clin. Invest. 129, 546–555. 10.1172/JCI121842 30561388 PMC6355218

[B39] Zhang C. Shang G. Gui X. Zhang X. Bai X. C. Chen Z. J. (2019). Structural basis of STING binding with and phosphorylation by TBK1. Nature 567, 394–398. 10.1038/s41586-019-1000-2 30842653 PMC6862768

[B40] Zhang X. Bai X. C. Chen Z. J. (2020). Structures and mechanisms in the cGAS-STING innate immunity pathway. Immunity 53, 43–53. 10.1016/j.immuni.2020.05.013 32668227

