# Supplementary material for: Licorice extract inhibits the cGAS-STING pathway and protects against non-alcoholic steatohepatitis
Source: Front Pharmacol. 2023 Apr 4;14:1160445. doi: 10.3389/fphar.2023.1160445 (PMC10111149; doi:10.3389/fphar.2023.1160445)
Supplement: Supplementary file 1 [file DataSheet1.pdf]

Supplementary Figure 1

Certificate of Analysis

Report on the Detection of Licorice

Product Name	licorice	
Latin Name	Glycyrrhiza uralensis Fisch	
Date of production	2022-02-15	
Batch Number	DST20220215	
Source	**The dried roots and rhizomes of the leguminous plant Glycyrrhiza uralensis Fisch.**	
Test Item	Specifications	Results
Appearance	The texture of the roots and rhizomes is relatively firm, some have branches, the outer skin is not rough, mostly gray-brown, and the pores on the skin are fine and not prominent	Conforms
Identification	In the chromatogram of the test sample, fluorescent spots of the same color appeared at the corresponding positions as those of the reference medicinal materials; and at the corresponding positions as those of the reference products in the chromatogram, orange-yellow fluorescent spots of the same color appeared.	Conforms
Content %	liquiritin≥0.5%	0.7%
	glycyrrhizic acid≥2%	2.13%
Loss on Drying %	≤5.0	6.25
Ash Content %	≤8.0	3.88
Residual Analysis:		
Heavy Metals PPM	≤10	Complies
(Pb) PPM	≤2	Complies
(As) PPM	≤2	Complies
Microbiological:		
Total Plate Count cfu/g	≤1000	Complies
Moulds&Yeast cfu/g	≤100	Complies
E.Coli cfu/g	Negative	Negative
Samonella cfu/g	Negative	Negative
Staph.aureus cuf/g	Negative	Negative
Conclusion:	Conforms to specification	

QA: Chong chen

SpecialSeal: 成都德思特生物技术有限公司 质检章

Tel：028-85003950 Fax：028-85589956
http://www.lemeitian.cn Add：No. 128, West Haiko Road, Wenjiang District, Chengdu, Sichuan, China.

Supplementary Figure 2

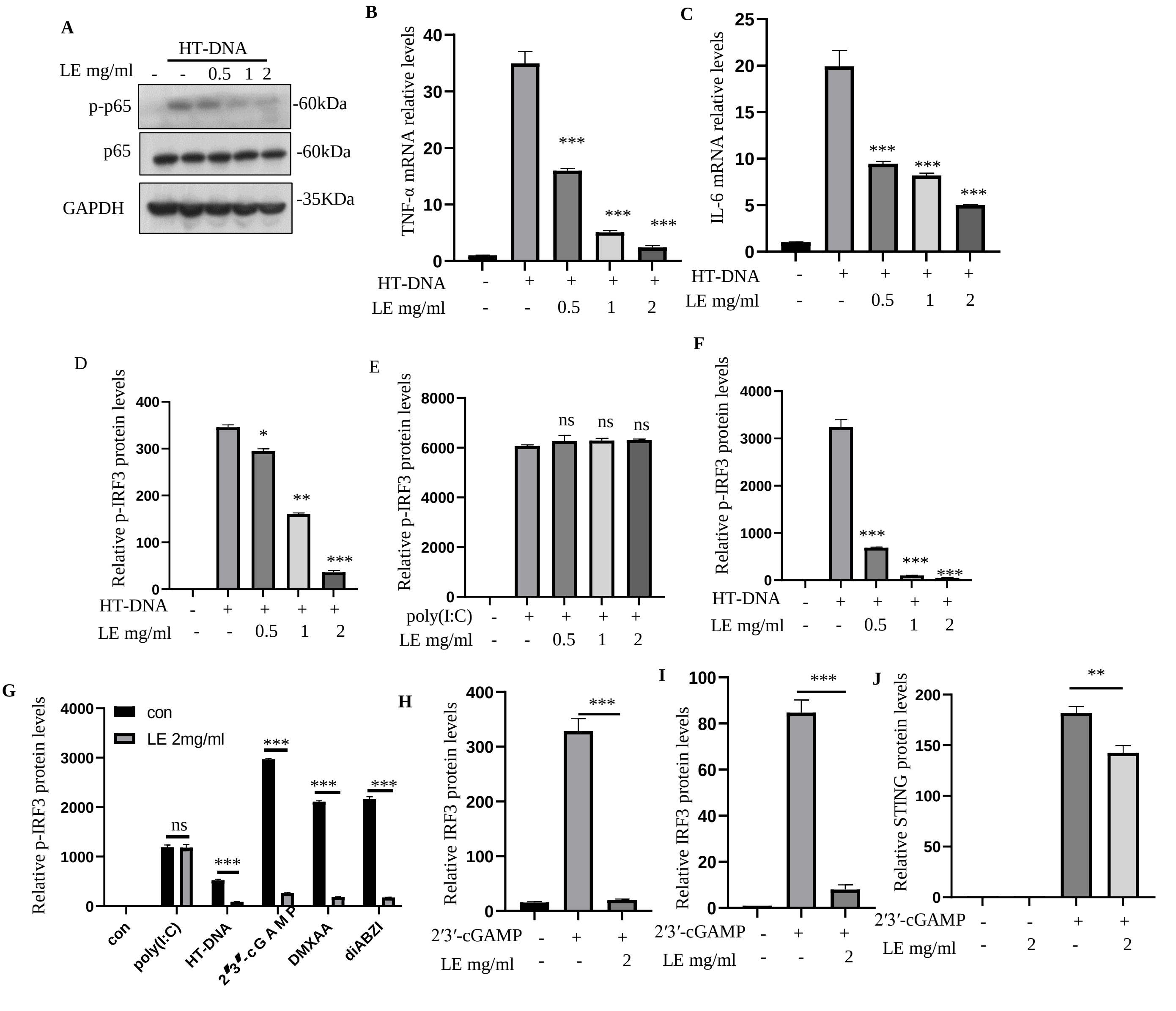

(A) BMDMs were pretreated for 1 hour with various concentrations of LE and then transfected with HT-DNA. Using Western blotting, p-p65, p65, and GAPDH were analyzed 2 hours after HT-DNA transfection. (B-C) Quantitative PCR was performed to measure the TNF-α,and IL-6 mRNA 4 hours after HT-DNA transfection. (D) Quantitative analysis of figure 1C. (E) Quantitative analysis of figure 1F. (F) Quantitative analysis of figure 1I. . (G) Quantitative analysis of figure 2A. (H) Quantitative analysis of figure 3A. (I) Quantitative analysis of figure 3B. (J) Quantitative analysis of figure 3F

Supplementary Figure 3

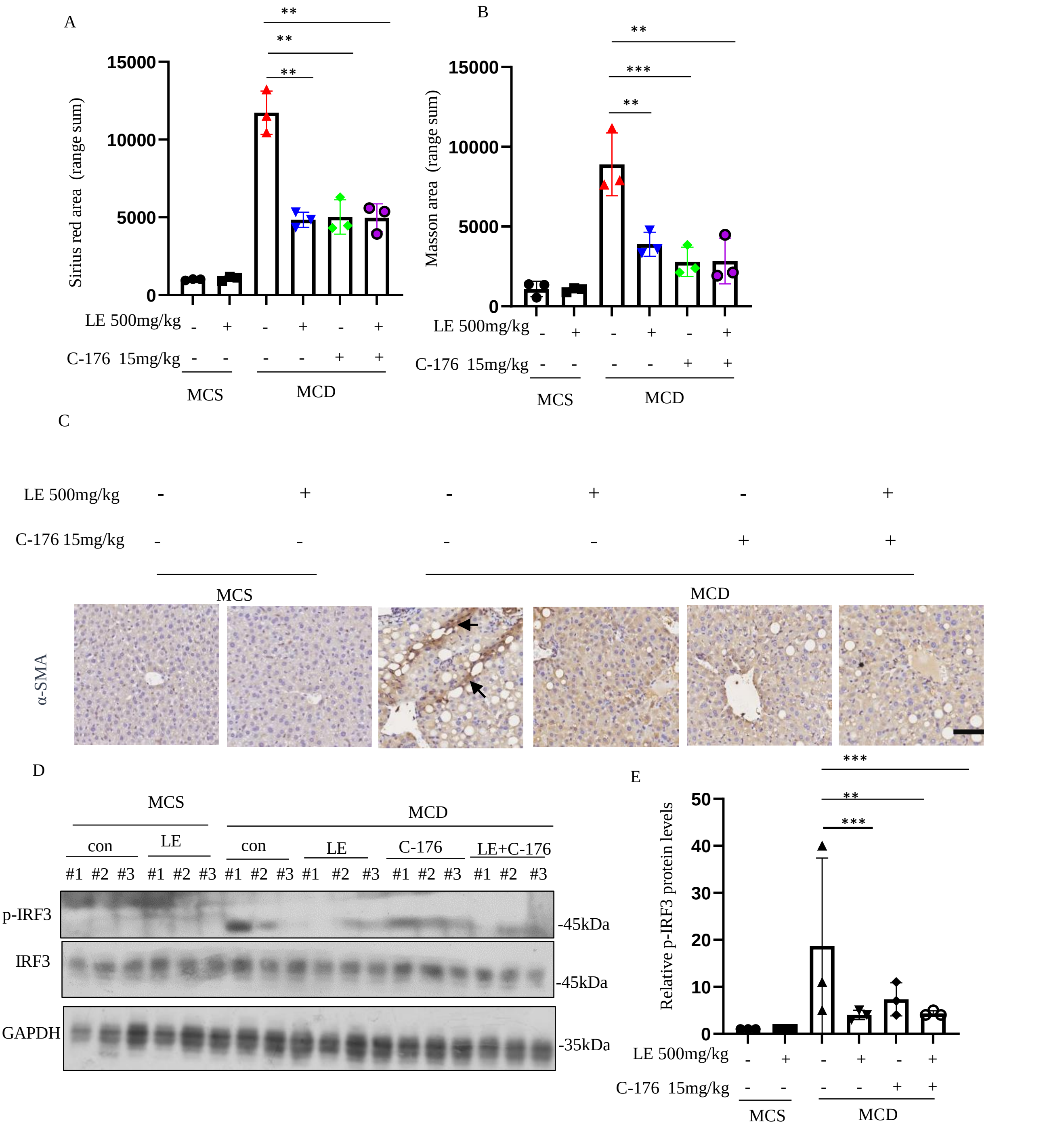

(A-B) Quantitative results of Sirius red (A) and Masson (B) .(C) Six weeks old female C57BL/6 mice were continuously fed MCS or MCD diets for 6 weeks under the same growth conditions. At the same time, they were administered with licorice extract by gavage, injected intraperitoneally with C-176（15mg/kg）, or treated with a combination of licorice extract and C-176 every two days (n = 8 mice per group). Representative immunohistochemical staining. images of α-SMA were shown. Scale bars represent 100 µm .(D) Liver lysates were examined for total and phosphorylated IRF3. (D) quantified the activated p-IRF3 using ImageJ software.

Supplementary Table

Table 1

Gene name	Forward primers	Reverse primers
Human *Ifnb*	TCCAAATTGCTCTCCTGTTG	GCAGTATTCAAGCCTCCCAT
Human β-actin	CATGTACGTTGCTATCCAGGC	CTCCTTAATGTCACGCACGAT
mIL-6	CACTTCACAAGTCGGAGGCT	CTGCAAGTGCATCATCGTTGT
mTNFα	CATCTTCTCAAAATTCGAGTGACAA	CCAGCTGCTCCTCCACTTG
mα-SMa	TCATCACCAACTGGGACGAC	GTCATTTTCTCCCGGTTGGC
Mouse *Ifnb*	TCCGAGCAGAGATCTTCAGGAA	TGCAACCACCACTCATTCTGAG
Mouse *Cxcl10*	GCCGTCATTTTCTGCCTCA	CGTCCTTGCGAGAGGGATC
Mouse *Isg15*	TGACTGTGAGAGCAAGCAGC	CCCCAGCATCTTCACCTTTA
Mouse collagen1	CTGGCGGTTCAGGTCCAAT	TTCCAGGCAATCCACGAGC
Mouse β-actin	TCCAAATTGCTCTCCTGTTG	CCAGTTGGTAACAATGCCATGT

Table 2

Histopathological score	ALL (n=48)	MCS		MCD			
		control (n=8)	LE (n=8)	control (n=8)	LE (n=8)	C-176 (n=8)	LE+C-176 (n=8)
Ballooning, n (%)					P=0.341	P=0.024	P=0.054
0	16(33.3)	8(100.0)	8(100.0)	0(0.0)	0(0.0)	0(0.0)	0(0.0)
1	19(39.6)	0(0.0)	0(0.0)	0(0.0)	5(62.5)	6(75.0)	7(87.5)
2	13(27.1)	0(0.0)	0(0.0)	8(100.0)	3(37.5)	2(25.0)	1(12.5)
Inflammation, n (%)					P=0.010	P=0.011	P<0.001
0	16(33.3)	8(100.0)	8(100.0)	0(0.0)	0(0.0)	0(0.0)	0(0.0)
1	9(18.8)	0(0.0)	0(0.0)	0(0.0)	3(37.5)	3(37.5)	2(25.0)
3	20(41.7)	0(0.0)	0(0.0)	6(75.0)	5(62.5)	4(50.0)	5(62.5)
4	3(6.3)	0(0.0)	0(0.0)	2(25.0)	0(0.0)	1(12.5)	1(12.5)
Steatosis, n (%)					P=0.228	P=0.861	P=0.051
0	16(33.3)	8(100.0)	8(100.0)	0(0.0)	0(0.0)	0(0.0)	0(0.0)
1	3(6.3)	0(0.0)	0(0.0)	0(0.0)	1(12.5)	1(12.5)	1(12.5)
2	23(47.9)	0(0.0)	0(0.0)	3(37.5)	6(75.0)	5(62.5)	6(75.0)
3	6(12.5)	0(0.0)	0(0.0)	5(62.5)	1(12.5)	2(25.0)	1(12.5)
Fibrosis, n (%)					P=0.008	P=0.007	P=0.025
0	16(33.3)	8(100.0)	8(100.0)	0(0.0)	0(0.0)	0(0.0)	0(0.0)
1a	2(4.2)	0(0.0)	0(0.0)	0(0.0)	0(0.0)	0(0.0)	2(25.0)
1b	3(6.3)	0(0.0)	0(0.0)	0(0.0)	1(12.5)	1(12.5)	1(12.5)
1c	7(14.6)	0(0.0)	0(0.0)	0(0.0)	3(37.5)	2(25.0)	2(25.0)
2	14(29.2)	0(0.0)	0(0.0)	2(25.0)	4(50.0)	5(62.5)	3(37.5)
3	4(8.3)	0(0.0)	0(0.0)	4(50.0)	0(0.0)	0(0.0)	0(0.0)
4	2(4.2)	0(0.0)	0(0.0)	2(25.0)	0(0.0)	0(0.0)	0(0.0)
